# Antibiotic use among SARI patients according to the AWaRe classification before and during the COVID-19 pandemic in Bangladesh

**DOI:** 10.1017/ash.2023.252

**Published:** 2023-09-29

**Authors:** Md Ariful Islam, Md. Zakiul Hassan, Mohammad Abdul Aleem, Zubair Akhtar, Tanzir Ahmed Shuvo, Md Kaousar Ahmmed, Syeda Mah-E-Muneer, Md Abdullah Al Jubayer Biswas, Ayesha Afrin, Probir Kumar Ghosh, Fahmida Chowdhury

## Abstract

**Background:** Irrational antibiotic use among hospitalized patients can lead to antibiotic resistance. For rational use, the WHO introduced the Access, Watch, and Reserve (AWaRe) classification of antibiotics. We explored antibiotic use according to the AWaRe classification among patients hospitalized with severe acute respiratory infection (SARI) between the prepandemic and COVID-19 pandemic periods in Bangladesh. **Methods:** From June 2017 to November 2022, we analyzed SARI inpatient data from the hospital-based influenza surveillance platform at 9 tertiary-level hospitals in Bangladesh. We defined June 2017–February 2020 as the prepandemic period and March 2020–November 2022 as the pandemic period. Physicians identified inpatients meeting the WHO SARI case definition and recorded patient demographics, clinical characteristics, and antibiotics received during hospitalization. We used descriptive statistics to summarize the data. **Results:** We enrolled 20,640 SARI patients (median age, 20 years; IQR, 1.6–50; 63% male); and among them, 18,197 (88%) received antibiotics (26% of those received >1 different course of antibiotics). Compared to the prepandemic period, the proportion of antibiotic use among SARI patients was higher during the pandemic: 93% (9,887 of 10,655) versus 83% (8,310 of 9,985) (*P* < .001). According to AWaRe classification, Access, Watch, and Reserve groups accounted for 32% (n = 2,623), 86% (n = 7,158), and 0.05% (n = 4), respectively, before the pandemic and 32% (n = 3,194), 90% (n = 8,850), and 0.08% (n = 8), respectively, during the pandemic (Fig.). The most common antibiotic prescribed for children aged <5 years during the prepandemic was ceftriaxone (n = 1,940, 74%), followed by amikacin (n = 325, 13%) and flucloxacillin (n = 300, 12%); similarly, during the pandemic, most common antibiotic prescribed was ceftriaxone (n = 3,097, 79%), followed by amikacin (n = 723, 18%) and flucloxacillin (n = 348, 9%). The most common antibiotic prescribed for patients aged ≥5 years during the prepandemic period was ceftriaxone (n = 3,174, 54%), followed by amoxicillin-clavulanic acid (n = 1,304, 22%) and azithromycin (n = 1,038, 18%). During the pandemic, the most common antibiotic prescribed for patients aged ≥5 years was ceftriaxone (n = 3,793, 64%), followed by amoxicillin-clavulanic acid (n = 1,327, 22%) and clarithromycin (n = 797, 13%). Among children aged <5 years, use of the Watch group of antibiotics during the prepandemic and pandemic periods was similar: 94% (n = 3,688) versus 95% (n = 2,347) (*P* = .099). However, among patients aged ≥5 years, the use of Watch antibiotics was higher during the pandemic compared to the prepandemic period: 87% (n = 5,163) versus 82% (n = 4,811) (*P* < .001). **Conclusions:** Use of antibiotics in the Watch group was predominant among SARI patients both before and during the COVID-19 pandemic, and it increased among SARI patients aged ≥5 years during the pandemic period in Bangladesh. Promoting antibiotic stewardship programs for physicians, including in-service training on antibiotic use, could reduce irrational antibiotic use, which might contribute to mitigating antibiotic resistance in the country.

**Figure:**
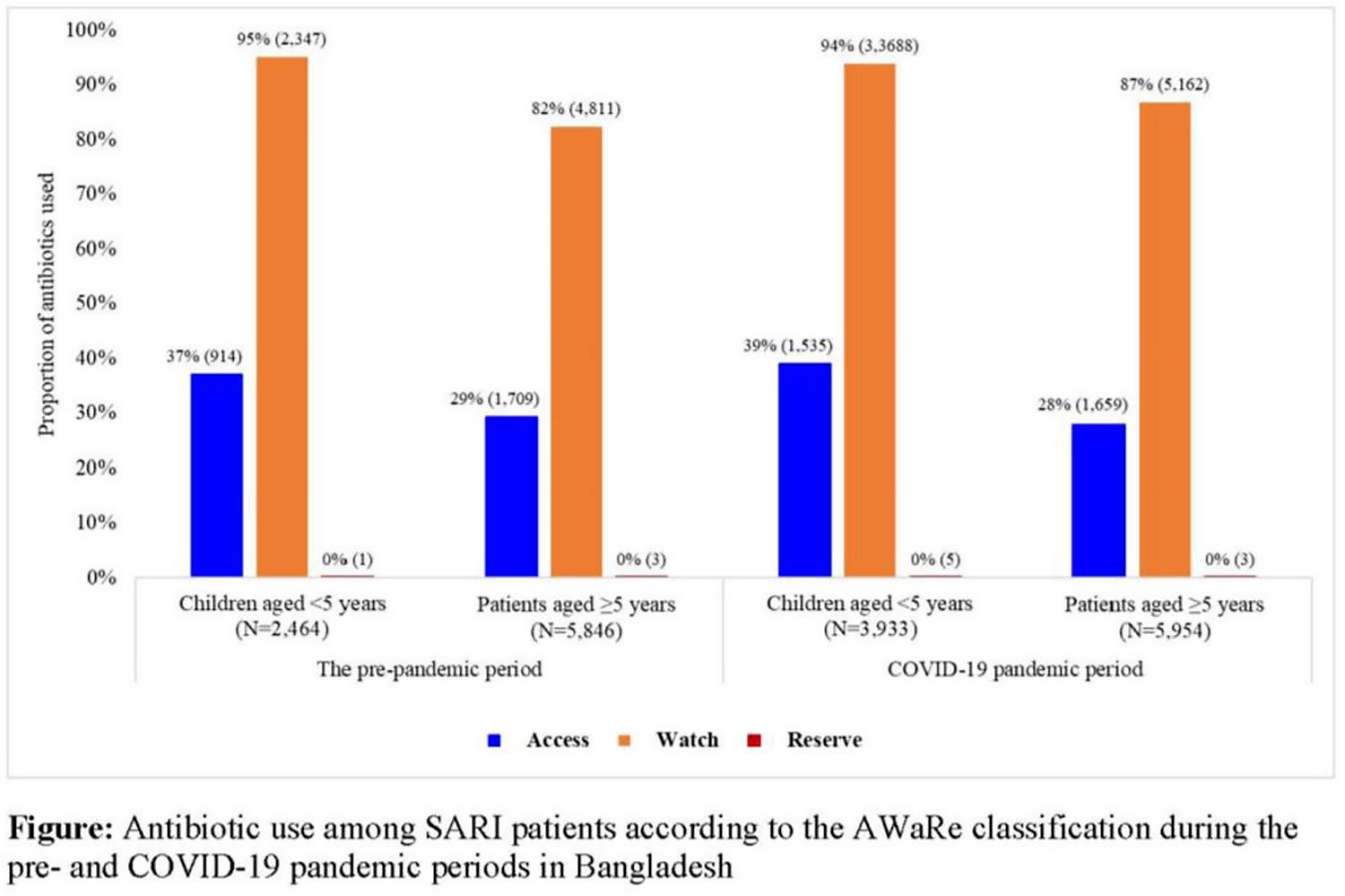

**Financial support:** This study was funded by the CDC.

**Disclosures:** None

